# Generation of CD20-specific TCRs for TCR gene therapy of CD20^low^ B-cell malignancies insusceptible to CD20-targeting antibodies

**DOI:** 10.18632/oncotarget.12778

**Published:** 2016-10-20

**Authors:** Lorenz Jahn, Dirk M. van der Steen, Renate S. Hagedoorn, Pleun Hombrink, Michel G.D. Kester, Marjolein P. Schoonakker, Daniëlle de Ridder, Peter A. van Veelen, J.H. Frederik Falkenburg, Mirjam H.M. Heemskerk

**Affiliations:** ^1^ Department of Hematology, Leiden University Medical Center, 2300 RC Leiden, The Netherlands; ^2^ Department of Hematopoiesis, Sanquin Research, 1006 AD Amsterdam, The Netherlands; ^3^ Department of Immunohematology and Blood Transfusion, Leiden University Medical Center, 2300 RC Leiden, The Netherlands; ^4^ Center for Proteomics and Metabolomics, Leiden University Medical Center, 2300 RC Leiden, The Netherlands

**Keywords:** CD20, TCR gene transfer, monoclonal antibodies, immunotherapy, B-cell leukemia and lymphoma

## Abstract

Immunotherapy of B-cell leukemia and lymphoma with CD20-targeting monoclonal antibodies (mAbs) has demonstrated clinical efficacy. However, the emergence of unresponsive disease due to low or absent cell surface CD20 urges the need to develop additional strategies. In contrast to mAbs, T-cells via their T-cell receptor (TCR) can recognize not only extracellular but also intracellular antigens in the context of HLA molecules. We hypothesized that T-cells equipped with high affinity CD20-targeting TCRs would be able to recognize B-cell malignancies even in the absence of extracellular CD20. We isolated CD8^+^ T-cell clones binding to peptide-MHC-tetramers composed of HLA-A*02:01 and CD20-derived peptide SLFLGILSV (CD20_SLF_) from HLA-A*02:01^neg^ healthy individuals to overcome tolerance towards self-antigens such as CD20. High avidity T-cell clones were identified that readily recognized and lysed primary HLA-A2^pos^ B-cell leukemia and lymphoma in the absence of reactivity against CD20-negative but HLA-A2^pos^ healthy hematopoietic and nonhematopoietic cells. The T-cell clone with highest avidity efficiently lysed malignant cell-lines that had insufficient extracellular CD20 to be targeted by CD20 mAbs. Transfer of this TCR installed potent CD20-specificity onto recipient T-cells and led to lysis of CD20^low^ malignant cell-lines. Moreover, our approach facilitates the generation of an off-the-shelf TCR library with broad applicability by targeting various HLA alleles. Using the same methodology, we isolated a T-cell clone that efficiently lysed primary HLA-B*07:02^pos^ B-cell malignancies by targeting another CD20-derived peptide. TCR gene transfer of high affinity CD20-specific TCRs can be a valuable addition to current treatment options for patients suffering from CD20^low^ B-cell malignancies.

## INTRODUCTION

Therapeutic monoclonal antibodies (mAb) such as rituximab and ofatumumab have demonstrated the clinical efficacy of targeting the B-cell restricted antigen CD20 for the treatment of B-cell lymphomas and leukemia. Although CD20 is also expressed on healthy B-cells which are depleted in the course of therapy, long-term B-cell aplasia is well manageable [1, 2]. However, refractory disease to CD20-targeted mAb treatment has been reported with various mechanisms of resistance: downregulation of CD20 expression [3–5], internalization of CD20:mAb complex [6], and inhibition of complement-dependent cytotoxicity (CDC) [7–10]. Therefore, additional therapeutic strategies are required.

T-cell receptor (TCR) gene transfer is an attractive strategy to equip T-cells with TCRs of defined antigen-specificity. Due to their high sensitivity for cognate antigen presented in human leukocyte antigen (HLA), TCRs can induce T-cell activation even when antigen abundance is very low [11–13]. Moreover, since HLA molecules sample the entire endogenous protein repertoire, also intracellular antigens can be presented on the cell surface and are accessible for recognition by TCRs. However, the broad application of TCR-based adoptive immunotherapy directed against self-antigens such as CD20 is hampered by lack of an effective immune response against CD20-derived peptides presented in the context of autologous (self) HLA molecules. T-cells carrying high-affinity TCRs reactive to such self-antigens are deleted by negative selection during thymic development to prevent auto-reactivity. An attractive strategy to target self-antigens is to exploit the immunogenicity of such antigens presented in the context of allogeneic (non-self) HLA. Presentation of self-antigens in the context of allogeneic HLA (alloHLA) can induce strong antigen-specific T-cell responses as observed in HLA-mismatched hematopoietic stem cell transplantation [14, 15]. We and others have developed several approaches to generate T-cell responses towards specific antigens from which individual T-cell clones and their TCRs can be isolated [16–20].

We hypothesized that high-affinity TCRs, targeting HLA-presented peptides derived from CD20, would be a valuable addition to current CD20-targeting therapies by providing means to target B-cell malignancies in which CD20 expression is insufficient for mAb-based approaches. To target CD20 in a TCR-based approach, we searched for high-affinity TCRs directed against the CD20-derived peptide SLFLGILSV (CD20_SLF_) that is endogenously processed and presented in the context of HLA-A*02:01 (HLA-A2) [21]. Furthermore, to broaden the applicability of a CD20-specific TCR-based approach, we searched for additional CD20-derived peptides in the HLA ligandome of B- lymphocytes [22], and were able to identify using a similar high-throughput screening method a high-avidity T-cell clone directed against the CD20-derived peptide RPKSNIVLL (CD20_RPK_) presented in HLA-B*07:02 (HLA-B7).

## RESULTS

### Identification of CD20-specific T-cell clones by high-throughput screening

We used peptide-MHC (pMHC)-tetramers composed of CD20-derived peptide SLFLGILSV (CD20_SLF_) bound to HLA-A2 for the isolation of T-cells reactive to CD20 from PBMCs of 6 healthy HLA-A2^neg^ individuals. Starting with 250 to 1,000 × 10^6^ PBMCs, cells binding to pMHC-tetramers were first enriched by magnetic-associated cell sorting (MACS). From the positive fractions, containing the pMHC-tetramer labelled cells, thousands of pMHC-tetramer^+^ CD8^+^ T-cells were single-cell sorted by fluorescent-activated cell sorting (FACS) and clonally expanded for two weeks. In total, 3,632 T-cell clones were isolated and expanded. The number of T-cell clones that could be isolated from an individual varied between 71 and 1,605 (Supplementary Table S1). In an initial high-throughput screen, all 3,632 clones were assessed for their specificity of CD20_SLF_ peptide by stimulation with HLA-A2^pos^ but CD20-negative K562 cells (K562-A2) that were either left untreated or pulsed with 50 nM CD20_SLF_. The secretion of both IFN-γ and GM-CSF was measured since a substantial number of pMHC-tetramer^pos^ T-cell clones has poor intrinsic IFN-γ production [23]. Therefore, using both cytokines as readout parameters allows for the assessment of more T-cell clones than using IFN-γ alone. Three reactivity patterns could be observed based on the secretion of GM-CSF and IFN-γ. For reasons of brevity and the additional value of GM-CSF over IFN-γ, only data for GM-CSF is shown. More than 55% of T-cell clones did not produce any cytokine upon stimulation with either unloaded or peptide-pulsed K562-A2 cells as exemplified by clone 265 (Supplementary Figure S1A–S1B). These data indicated insufficient avidity to recognize HLA-bound CD20_SLF_. In contrast, many T-cell clones like 5F9 demonstrated reactivity towards HLA-A2^pos^ K562-A2 cells irrespective of absence or presence of CD20_SLF_ peptide, suggesting a lack of specificity for peptide CD20_SLF_. Around 38.4% of all tested T-cell clones showed such a reactivity profile in the initial high-throughput screen (Supplementary Figure S1A–S1B). However, 213 T-cell clones, accounting for 5.8% of all tested T-cell clones, were selected that demonstrated peptide specificity by recognizing peptide-pulsed K562-A2 more robustly than unloaded K562-A2 and binding to pMHC-tetramer CD20_SLF_:A2. Supplementary Table S1 summarizes the distributions of T-cell clones per reactivity profile for each of the 6 HLA-A2^neg^ healthy individuals from which the T-cell clones were isolated.

**Figure 1:**
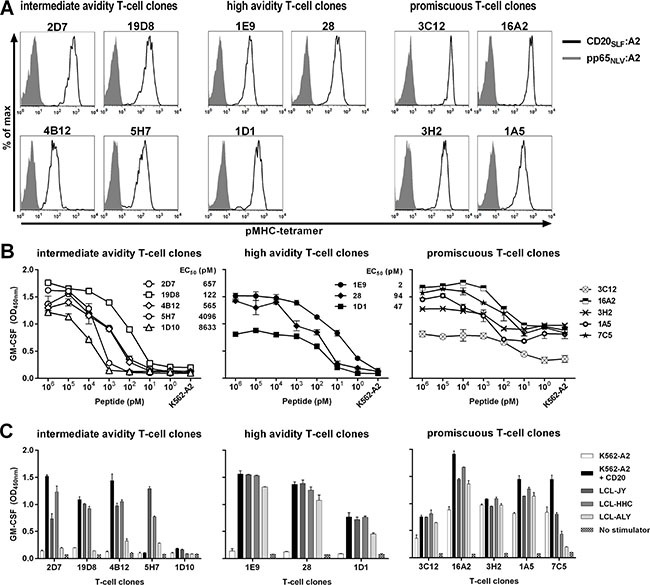
Identification of high-avidity peptide-specific T-cell clones from selected candidate T-cell clones Shown are data of representative clones among T-cell clones that were selected following high-throughput screening. (**A**) Shown are histograms of representative T-cell clones that were stained either with pMHC-tetramer composed of CD20_SLF_:A2 (black line) or a control pMHC-tetramer composed of CMV-derived peptide pp65_NLV_ bound to HLA-A2 (pp65_NLV_:A2, grey area). See Supplementary Figure S2 for additional T-cell clones and controls. (**B**) T-cell clones were cocultured with CD20^neg^ K562-A2 cells pulsed with titrated amounts of peptide CD20_SLF_ or left untreated (K562-A2). Values behind T-cell clones indicate peptide concentration at half maximum cytokine production (EC_50_). (**C**) T-cell clones were cocultured with K562-A2 cells or K562-A2 cell transduced to express CD20 (K562-A2 + CD20) or 3 HLA-A2^pos^ B-LCLs that endogenously express CD20 (LCL-JY, LCL-HHC, and LCL-ALY). After 18 hours of coculture supernatant was harvested and GM-CSF production was assessed using standard ELISA. Shown are means and standard deviations of one representative experiment carried out in duplicate.

Next, we continued only with the assessment of the 213 selected T-cell clones that demonstrated specificity for peptide CD20_SLF_ in the initial screening. First, specific binding to pMHC-tetramer CD20_SLF_:A2 was validated for these T-cell clones. All 213 selected T-cell clones stained with pMHC-tetramer CD20_SLF_:A2 although the intensity of the staining was variable between clones (Figure 1A and Supplementary Figure S2). Staining with pMHC-tetramer CD20_SLF_:A2 was specific for all 213 T-cell clones since no binding to a control pMHC-tetramer composed of HLA-A2 and peptide NLVPMVATV derived from the human cytomegalovirus (CMV) protein pp65 (pp65_NLV_) was observed (Figure 1A and Supplementary Figure S2).

Next, we aimed to identify T-cell clones with high peptide sensitivity and sufficient avidity to recognize endogenously processed peptide. The selected clones demonstrated various degrees of peptide sensitivity when stimulated with titrated amounts of CD20_SLF_ peptide loaded on K562-A2 cells. For many T-cell clones of intermediate avidity, micro- to nanomolar amounts of exogenously loaded CD20_SLF_ were required to induce cytokine production (Figure 1B left). This absence of high peptide sensitivity also translated to poor recognition of endogenously processed peptide, measured by recognition of three HLA-A2^pos^ CD20-expressing Epstein-Barr virus (EBV)-transformed B-lymphoblastic cell-lines (B-LCLs) or K562-A2 cells stably transduced to express CD20 (Figure 1C left). Intermediate avidity T-cell clones either failed to recognize any or reacted only towards a subset of CD20-expressing stimulators. On the other hand, high avidity T-cell clones such as 1E9, 28, and 1D1 demonstrated peptide sensitivities ranging from the nano- to picomolar range (Figure 1B middle). Higher sensitivity to exogenously loaded peptide correlated with the capacity to recognize endogenously processed peptide. High-avidity clones 1E9, 28 and 1D1 readily recognized all three B-LCLs and CD20-transduced K562-A2 cells (Figure 1C middle).

In contrast, many of the T-cell clones lacked exclusive specificity for peptide CD20_SLF_. This subset of promiscuous T-cell clones recognized peptide-loaded K562-A2 in a peptide concentration-dependent manner (Figure 1B right). However, cytokine production could also be observed towards unloaded K562-A2, albeit to a lesser extent (Figure 1B right and 1C right). These data most likely indicated that besides peptide CD20_SLF_ also other unknown peptides could be recognized in the context of HLA-A2. Similar data was obtained measuring IFN-γ secretion in all experiments (data not shown).

In conclusion, we isolated T-cell clones specific for peptide CD20_SLF_ presented in HLA-A2 from the allorepertoire of HLA-A2^neg^ individuals. Only the most sensitive clones demonstrated sufficient avidity to robustly recognize endogenously processed peptide on all CD20-expressing stimulator cells tested.

### Robust recognition of HLA-A2^pos^ ALL cell-lines by high-avidity T-cell clones

**Figure 2:**
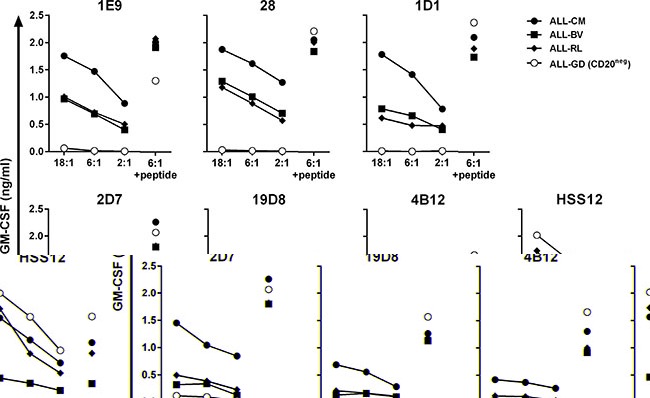
Recognition of malignant cell samples by high-avidity T-cell clones T-cell clones were cocultured with HLA-A2^pos^ ALL cell-lines at indicated stimulator-to-responder ratios. Stimulator cell-line ALL-GD did not express CD20 as measured by RT-qPCR (Figure 3C). Controls included stimulators pulsed with 50 nM peptide CD20_SLF_ (+ peptide) and T-cell clone HSS12 recognizing peptide FTWEGLYNV from the ubiquitously expressed gene USP11 in the context of HLA-A2 to confirm HLA-A2 expression on the ALL cell-lines. Cytokine production was assessed after 18 hours of coincubation. Representative data from one of three independent experiments.

Next, we examined the recognition of HLA-A2^pos^ ALL cell-lines by a subset of CD20-specific T-cell clones. High-avidity T-cell clones 1E9 and 28 demonstrated robust recognition of 3 ALL cell-lines ALL-CM, ALL-BV and ALL-RL (Figure 2). Also clone 1D1 recognized all 3 ALL cell-lines, although to a lesser extent. In contrast, intermediate avidity T-cell clones 2D7, 19D8 and 4B12 only weakly recognized or failed to show any reactivity towards the ALL cell-lines. The degree of reactivity closely matched the observed peptide sensitivities. HLA-A2^pos^ but CD20-negative ALL cell-line ALL-GD could not be recognized by any clone.

In summary, T-cell clones demonstrating highest peptide sensitivity also more robustly recognized HLA-A2^pos^ CD20-expressing ALL cell-lines whereas intermediate avidity clones failed to consistently react to these stimulators.

### Efficient recognition of CD20^low^ B-cell malignancies

**Figure 3:**
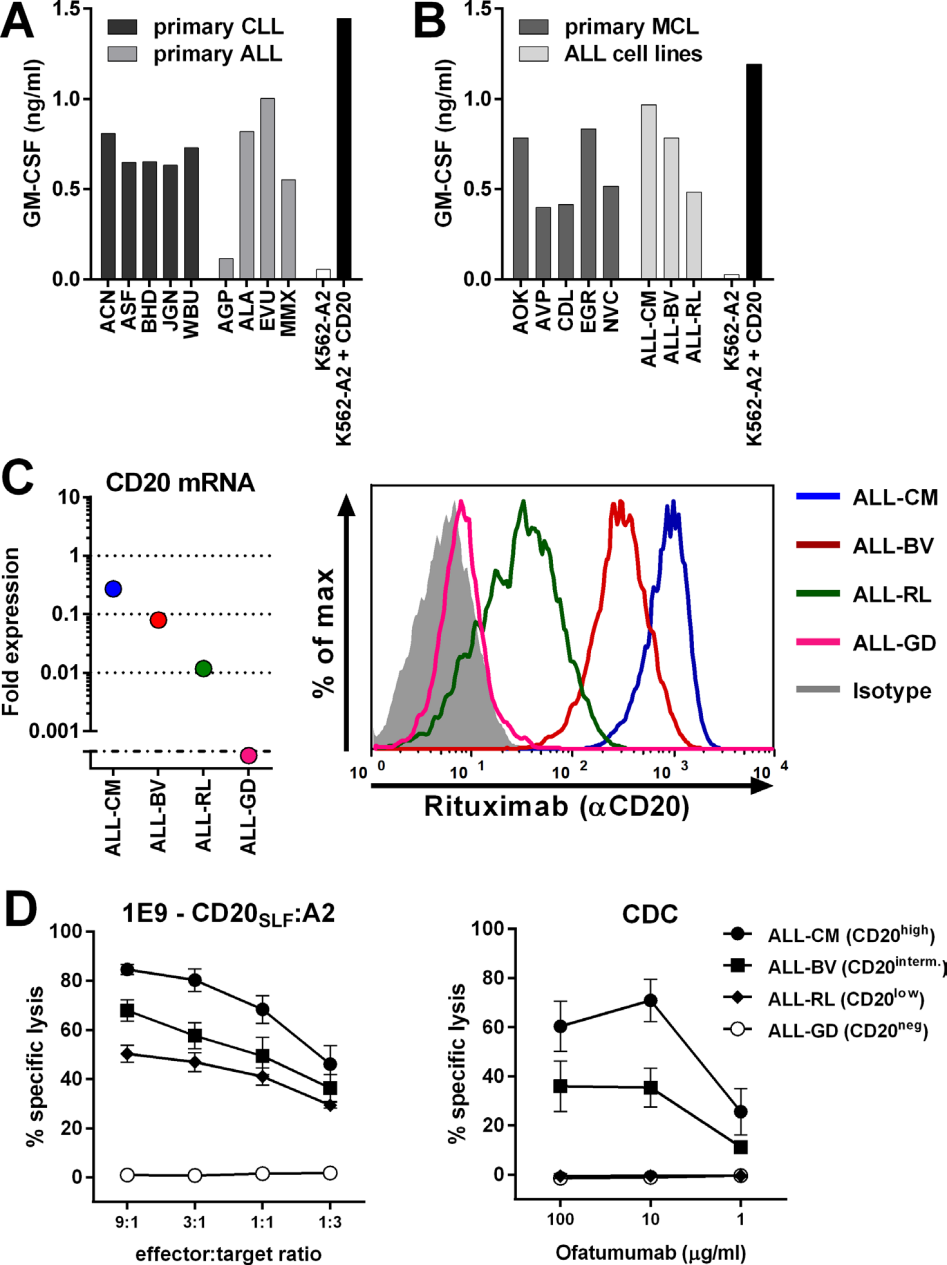
CD20-reactive T-cell clone 1E9 efficiently lyses CD20^low^ B-cell malignancies unsusceptible to CD20-targeting antibodies T-cell clone 1E9 having shown highest sensitivity for peptide CD20_SLF_ was tested for its recognition of various B-cell malignancies. (**A**–**B**) T-cell clone 1E9 was cocultured with HLA-A2^pos^ primary B-cell malignancies or ALL cell-lines. Primary samples included chronic lymphocytic leukemia (CLL) and acute lymphoblastic leukemia (ALL) (A) or primary mantle cell lymphoma (MCL) (B). Primary ALL sample AGP did not demonstrate CD20 expression at the mRNA level (Supplementary Figure S4). Controls included CD20^neg^ K562-A2 and CD20-transduced K562-A2 (K562-A2 + CD20). Supernatant was harvested after 18 hours of coincubation and cytokine production was assessed by standard ELISA. Experiments were carried out in duplicate. One representative experiment of two independent experiments is shown. (**C**) On the left, expressions of CD20 mRNA in ALL cell-lines was measured by RT-qPCR and is shown as fold expression of average expression in healthy B-cells which was set to 1. The detection limit of the assay was > 0.001 fold. On the right, cell surface CD20 on ALL cell-lines was assessed by FITC-conjugated CD20-specific monoclonal antibody rituximab. Controls included staining with an isotype-matched antibody. (**D**) Cytotoxicity of clone 1E9 (left panel) or complement dependent cytotoxicity (CDC, right panel) was measured by standard Cr^51^-release assay. Cr^51^-labelled targets were incubated with clone 1E9 at different effector-to-target ratios or different concentrations of CD20-targeting monoclonal antibody ofatumumab in the presence of human serum for 5 hours. Targets include HLA-A2^pos^ ALL cell-lines ALL-CM, ALL-BV, ALL-RL and ALL-GD. Controls included incubation with T-cell clone HSS12, recognizing peptide FTWEGLYNV from the ubiquitously expressed gene USP11 and a CMV-reactive T-cell clone CTN (Supplementary Figure S6). Shown are means with standard deviations of one experiment performed in triplicate. CDC shows pooled data using human serum from three different individuals.

To investigate the clinical potential of high-avidity T-cell clones 1E9 and 28, we assessed their capacity to recognize primary HLA-A2^pos^ B-cell malignancies including chronic lymphocytic leukemia (CLL), acute lymphoblastic leukemia (ALL) and mantle cell lymphoma (MCL). Furthermore, to assess the sensitivity of our T-cell clones, we included primary malignant cell samples with varying degrees of CD20 mRNA expression in our analysis, including CD20^low^ samples with more than 33-fold reduced CD20 mRNA expression compared to healthy B cells. Both clones efficiently recognized all 5 primary CLL samples (Figure 3A and Supplementary Figure S3A). Recognition of all CLL samples was comparable although expression of CD20 mRNA varied greatly between samples (Supplementary Figure S4). Primary CLL sample ACN had a 6-fold reduced CD20 mRNA expression compared to healthy B-cells. In contrast, CLL sample JGN had a 100-fold reduction in CD20 expression but was still sufficiently recognized. Furthermore, 3 out of 4 primary ALL samples were readily recognized by both T-cell clones. Real-time quantitative PCR (RT-qPCR) revealed lack of CD20 mRNA expression in the unrecognized primary ALL sample AGP (Supplementary Figure S4). Strong recognition of CD20^low^ expressing ALL sample MMX was observed, which had a 40-fold reduction in CD20 expression compared to healthy B-cells. Additionally, both T-cell clones readily recognized all 5 tested primary MCL samples (Figure 3B and Supplementary Figure S3B). Of note, clones 1D1, 2D7, 19D8 and 4B12 having demonstrated lower sensitivities for peptide CD20_SLF_ also recognized primary B-cell malignancies, however, to a lower degree than clones 1E9 and 28 (Supplementary Figure S5). High reactivity towards 3 HLA-A2^pos^ ALL cell-lines was observed for clone 1E9 and 28 (Figure 3B and Supplementary Figure S3B). CD20 mRNA expression varied in these cell-lines by orders of magnitude which directly translated to lower CD20 cell surface expression in CD20^low^ expressing cells (Figure 3C). Although CD20 cell surface expression was drastically reduced, clone 1E9 still efficiently lysed CD20^low^ ALL-RL (Figure 3D). In contrast, the CD20-targeting monoclonal antibody ofatumumab did not induce lysis of ALL-RL by complement dependent cytotoxicity (CDC), whereas ALL-CM and ALL-BV were lysed (Figure 3D). Ofatumumab was used over rituximab due its increased induction of CDC [24]. HLA-A2^pos^ ALL-GD, which lacks CD20 mRNA and cell surface expression, could not be lysed by either T-cell clone 1E9 or CDC mediated by mAb ofatumumab.

These data demonstrated that CD20-reactive T-cell clones 1E9 and 28 potently recognized primary HLA-A2^pos^ B-cell malignancies including CLL, ALL and MCL. Additionally, primary cell samples and cell-lines showing hundred-fold reduced CD20 expression and reduced CD20 cell surface expression could still be efficiently lysed by T-cell clone 1E9 whereas CDC after incubation with CD20-targeting antibodies was absent.

### Reactivity of CD20-reactive T-cell clones is restricted to the B-cell compartment

**Figure 4:**
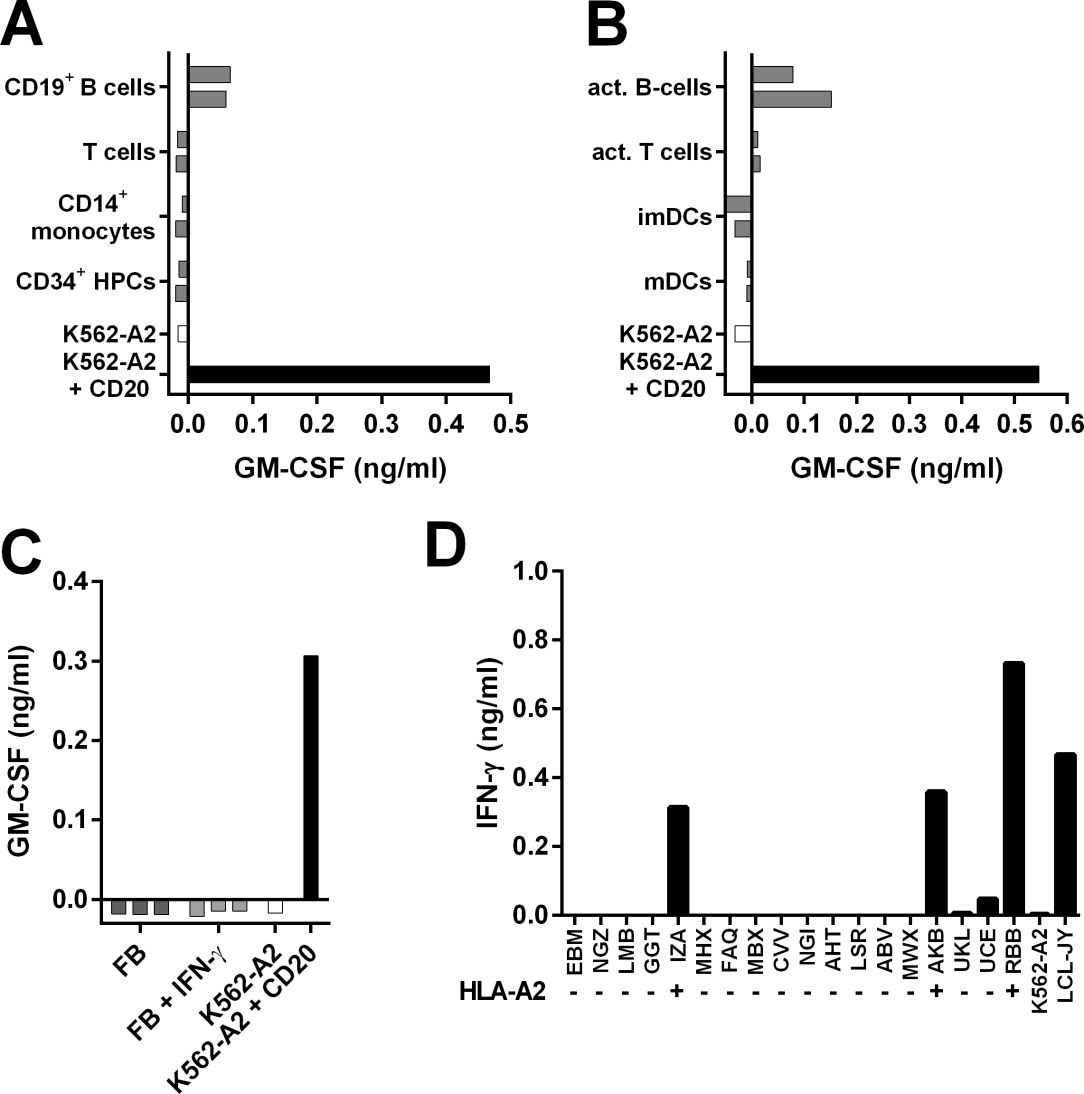
T-cell clone 1E9 demonstrates B-cell-restricted reactivity profile (**A**–**C**) T-cell clone 1E9 was cocultured with different cell subsets of hematopoietic (A–B) or nonhematopoietic (C) origin derived from HLA-A2^pos^ healthy individuals. Clone 1E9 was coincubated with primary (A) or activated (B) hematopoietic cell subsets from two different donors. Activated B-cells were generated by stimulating primary B-cell with CD40 ligand, activated T-cells were generated by stimulation with PHA. Immature and mature dendritic cells (imDCs and mDCs, respectively) were monocyte derived. Clone 1E9 was coincubated with fibroblasts (FB) that had been cultured either in the absence or presence of 200 IU/ml IFN-γ (+IFN-γ) for 4 days prior to the experiment (C). (**D**) Clone 1E9 was coincubated with a panel of B-LCLs expressing different HLA class I and class II alleles. HLA status regarding presence (+) or absence (−) of HLA-A*0201 is indicated. For a complete list of HLA genotype of all B-LCL see Supplementary Table S1. After 18 hours of coincubation, supernatant was harvested and cytokine production was assessed using standard ELISA. Shown are representative results of two independent experiments.

Having established potent reactivity towards B-cell malignancies, we next investigated the safety profile of clones 1E9 and 28 by stimulating them with a panel of HLA-A2^pos^ hematopoietic and nonhematopoietic cell subsets. Clones 1E9 and 28 did not recognize CD20^neg^ primary T-cells, CD14^+^ monocytes or CD34^+^ hematopoietic progenitor cells from 2 different individuals (Figure 4A and Supplementary Figure S3C). Moreover, no reactivity against activated T-cells or monocyte-derived immature and mature dendritic cells (DCs) from 2 individuals was observed (Figure 4B and Supplementary Figure S3D). However, CD20-expressing primary and CD40L-stimulated B-cells from both individuals were readily recognized. No reactivity towards 3 HLA-A2^pos^ fibroblasts was observed, even when fibroblasts were pre-treated with 200 IU/ml IFN-γ to simulate inflammation (Figure 4C and Supplementary Figure S3E).

To test whether clones 1E9 and 28 would be cross-reactive with HLA molecules other than HLA-A2, both clones were stimulated with a panel of B-LCLs expressing 95% of common and rare HLA class I alleles (Supplementary Table S2) [25]. Both clones reacted only towards HLA-A2^pos^ B-LCLs (Figure 4D and Supplementary Figure S3F) indicating no cross-reactivity with other tested HLA class I molecules.

In summary, both T-cell clones followed a CD20-restricted recognition profile correlating with CD20 expression. Reactivity was confined to the B-cell compartment and absent for other hematopoietic and nonhematopoietic cell subsets. No cross-reactivity with other HLA class I alleles was observed for either clone.

### TCR gene transfer installs potent CD20-specific reactivity onto recipient T-cells

**Figure 5:**
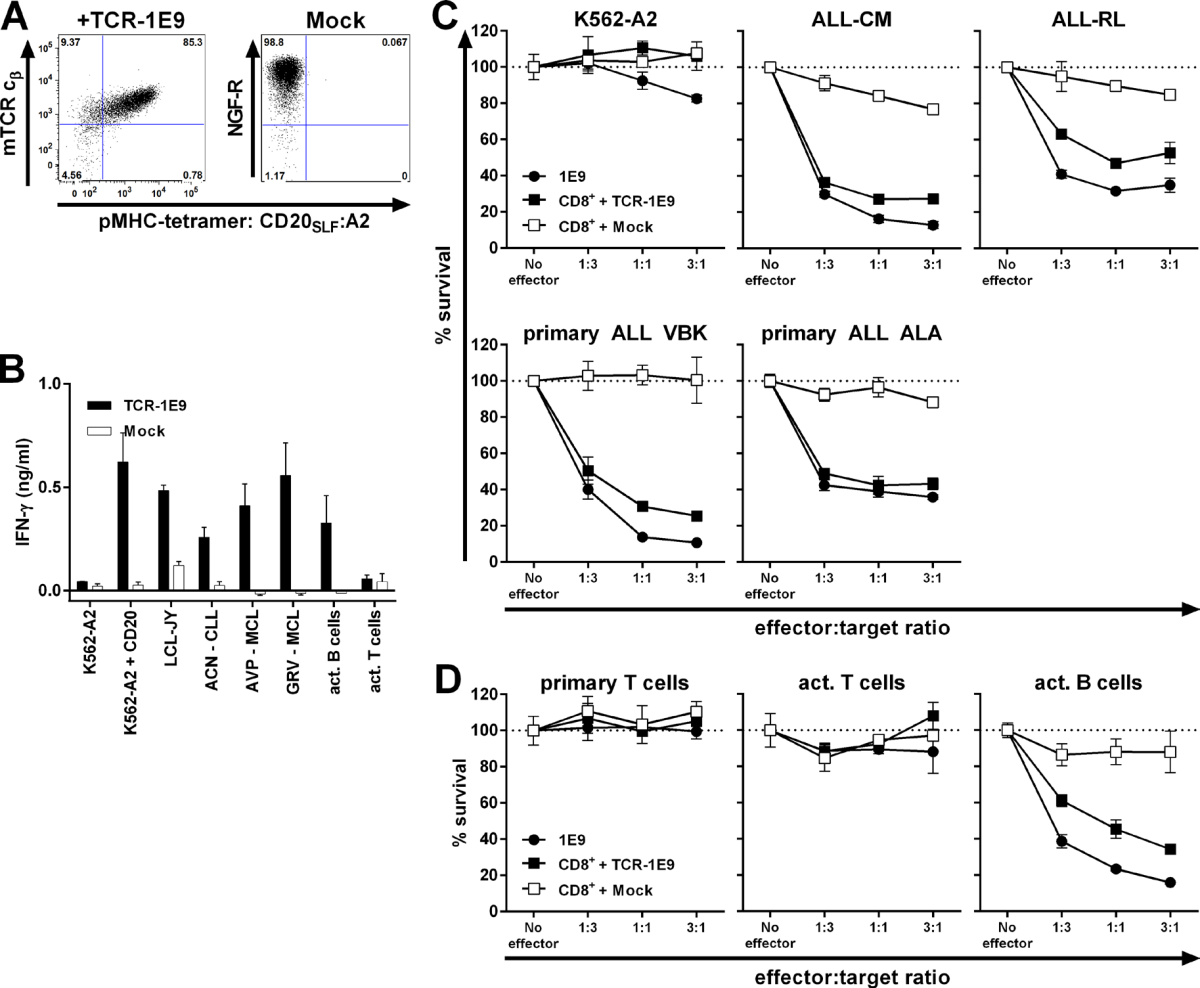
TCR gene transfer installs potent CD20-specific reactivity onto recipient CD8^+^ T-cells CD8^+^ T-cells from an HLA-A2^pos^ healthy individual were retrovirally transduced to express TCR of clone 1E9 (TCR-1E9). V(D)J segments of the TCRα and β chains were fused to the murine constant TCR domains. T-cells transduced with an empty vector (Mock) containing marker gene NGF-R served as control. (**A**) FACS plots of TCR or mock-transduced CD8^+^ T-cells following enrichment of transduced cells. TCR-transduced cells were enriched and stained by an antibody binding to the murine constant domain of the TCR β chain (mTCR c_β_) of the introduced TCR. Mock-transduced cells were isolated and stained with an antibody specific for marker gene NGF-R. All populations were incubated with pMHC-tetramer CD20_SLF_:A2. Numbers in corners indicate percentage cells in each quadrant. Dot plots are shown with bi-exponential axes. (**B**) TCR or mock-transduced T-cells were cocultured with different HLA-A2^pos^ stimulator cells. Primary cell samples include chronic lymphocytic leukemia (ACN) and mantle cell lymphomas (AVP and GRV). Activated B and T-cells were of autologous origin. Controls included CD20^neg^ K562-A2, CD20-transduced K562-A2 (K562-A2 + CD20) and CD20-expressing LCL-JY. IFN-γ production was assessed 18 hours after coculture. Experiment was performed in duplicate. Shown are means with standard deviations. (**C**–**D**) Survival of PKH-labelled targets cells was assessed after coculture with either T-cell clone 1E9 or TCR or mock-transduced T-cells at different effector-to-target ratios. Following 18 hours of coincubation, surviving targets cells were counted using FACS analysis and percent surviving cells was calculated. Malignant cell samples include ALL cell-lines ALL-CM and ALL-RL, and primary ALL samples VBK and ALA (C) Primary and activated T-cells and activated B-cells were of same origin as transduced CD8^+^ T-cells (D). Shown are means with standard deviations of one experiment performed in triplicate.

To test whether CD20-reactivity could be installed onto recipient T-cells by TCR gene transfer, we sequenced and cloned the TCR of clone 1E9 (TCR-1E9). We chose clone 1E9 because of its highest peptide sensitivity and consistent recognition of B-cell malignancies. The V(D) J segments of the TCRα and TCRβ chain were fused to murine TCR α and β constant domains on a modified MP71-TCR-flex retroviral backbone in order to induce preferential pairing and high expression of the introduced TCR chains [26]. The murine constant domain allowed for the isolation of TCR-transduced T-cells by staining with a murine TCR specifc antibody followed by isolation using MACS (Figure 5A). Transduced CD8^+^ T-cells of HLA-A2^pos^ healthy individuals expressed TCR-1E9 on the cell surface and stained highly with pMHC-tetramer CD20_SLF_:A2 whereas mock-transduced T-cells did not bind to pMHC-tetramer. TCR-transduced but not mock-transduced T-cells were able to efficiently recognize CD20-expressing HLA-A2^pos^ target cells (Figure 5B). TCR-modified CD8^+^ T-cells readily recognized primary CLL and MCL samples and autologous activated B-cells, whereas autologous activated T-cells were not recognized. Furthermore, we demonstrate in Figure 5C that CD20-TCR-transduced T-cells efficiently lysed ALL cell-lines, including ALL-RL which demonstrated reduced CD20-expression and insufficient cell surface CD20 to be targeted by monoclonal antibodies. Additionally, TCR-modified but not mock-transduced T-cells efficiently lysed primary ALL samples at very low effector-to-target ratios. Activated B-cells from an autologous source were also lysed by TCR-modified T-cells at levels comparable to parental clone 1E9 (Figure 5D). No lysis of neither primary nor activated autologous T-cells was observed, demonstrating the CD20-specificity and a safe reactivity profile of CD20-TCR-modified T-cells.

In conclusion, potent CD20-reactivity and specificity could be installed by TCR gene transfer. TCR-transduced T-cells readily recognized and lysed CD20-expressing B-cell malignancies, including samples with low CD20 expression. No off-target toxicity of CD20-negative targets was observed.

### Measured avidity for T-cell clones depends on expression of high-affinity TCR

**Figure 6:**
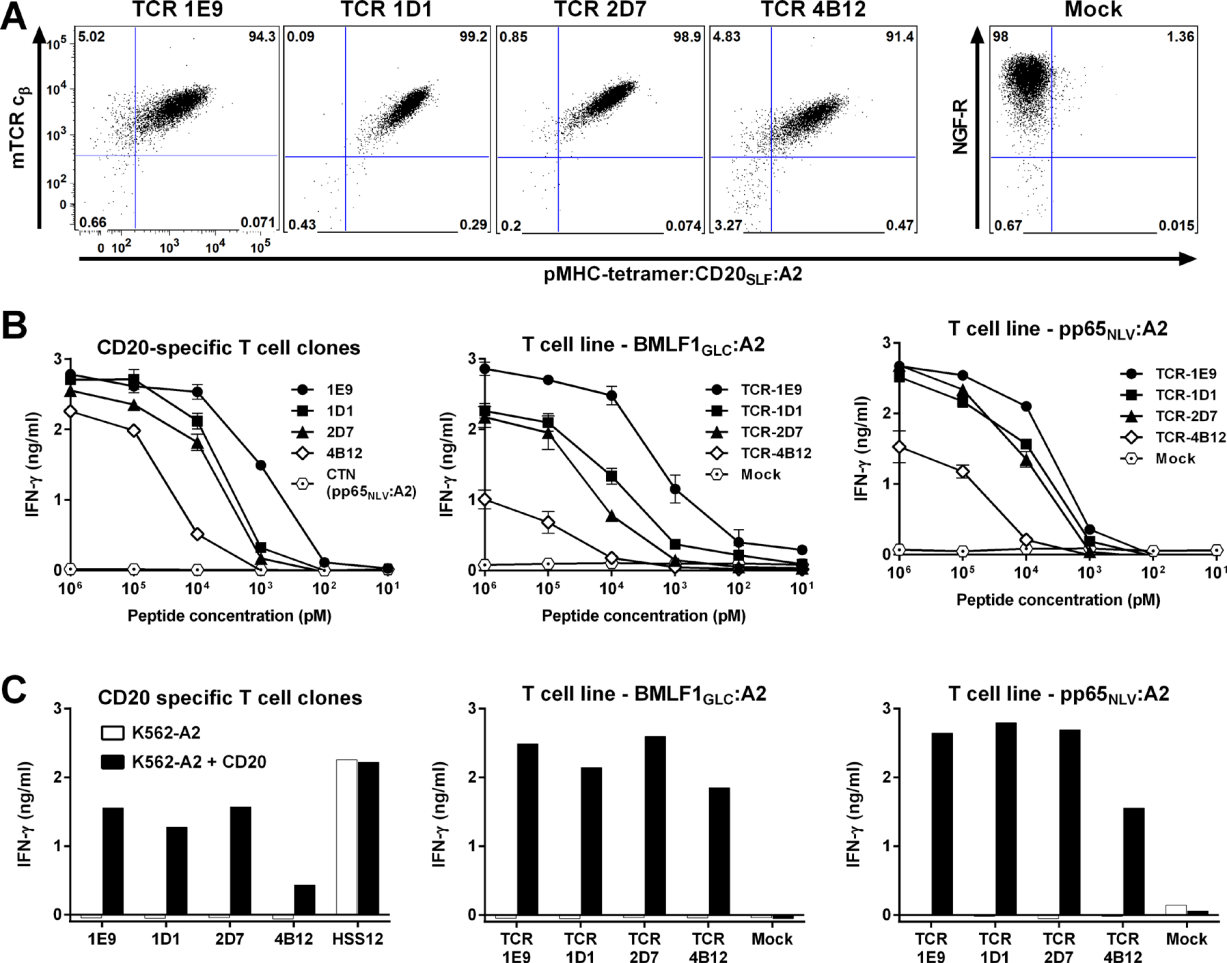
Functional avidity measured of T-cell clones depends on affinity of TCR Two virus-specific T-cell lines (BMLF1_GLC_:A2 or pp65_NLV_:A2) were retrovirally transduced to express the TCRs of clone 1E9, 1D1, 2D7 or 4B12 (TCR-1E9, TCR-1D1, TCR-2D7 or TCR-4B12, respectively). Transduction with an empty backbone (Mock) containing only the marker gene NGF-R served as control. (**A**) Shown are FACS plots of enriched TCR-transduced T-cells stained with an antibody specific for the murine constant domain of the TCR β chain (mTCR c_β_) of the introduced TCR. Cells were also stained with pMHC-tetramer CD20_SLF_:A2. Mock-transduced T-cells were stained with an antibody specific for marker gene NGF-R. Dot plots are shown with bi-exponential axes. (**B**) Original T-cell clones (left) or two virus-specific T-cell lines (middle and right) transduced with the different TCR constructs were coincubated with K562-A2 cells pulsed with titrated amounts of peptide CD20_SLF_. Controls included T-cell clone CTN specific for CMV-derived peptide pp65_NLV_. Shown are means with standard deviations of one experiment performed in duplicate. (**C**) Original T-cell clones (left) or two virus-specific T-cell lines (middle and right) transduced with the different TCR constructs were coincubated with K562-A2 cells or CD20-transduced K562-A2 (K562-A2 + CD20). USP11_FTW_-specific T-cell clone HSS12 served as control. IFN-γ production was assessed after 18 hours of coincubation. Shown are representative results of one of two independent experiments.

Before moving to the TCR gene transfer stage, candidate TCRs were primarily selected based on functional data obtained in experiments using the original T-cell clones. Therefore, quality of a TCR was assessed through functional avidity of that particular clone originating from either the naïve or memory compartment. To test whether intrinsic features of T-cell clones could have influenced the selection, we also cloned three additional TCRs for TCR gene transfer. Besides TCR-1E9, we selected the TCRs from clones 1D1, 2D7 and 4B12, since these clones demonstrated varying avidities. All four TCRs were efficiently expressed after transduction into CMV-specific T-cells and could be enriched to great purity (Figure 6A). Sensitivity of the TCR-transduced T-cells for titrated amounts of peptide CD20_SLF_ depended on the introduced TCR (Figure 6B–6C). T-cell transduced with TCR-1E9 were most sensitive. A gradual decrease in sensitivity was observed for T-cells transduced with TCR-1D1, TCR-2D7 and TCR-4B12. Peptide sensitivities of transduced T-cells closely mirrored peptide sensitivities observed for the parental T-cell clones.

These data indicated that screening for candidate TCRs is feasible on clonal populations of T-cells. Observed functional avidities of T-cell clones reflected the affinity of the TCRs and were less likely to be influenced by intrinsic features of each individual clone.

### Building a TCR library targeting various CD20-derived peptides and HLA-alleles

**Figure 7:**
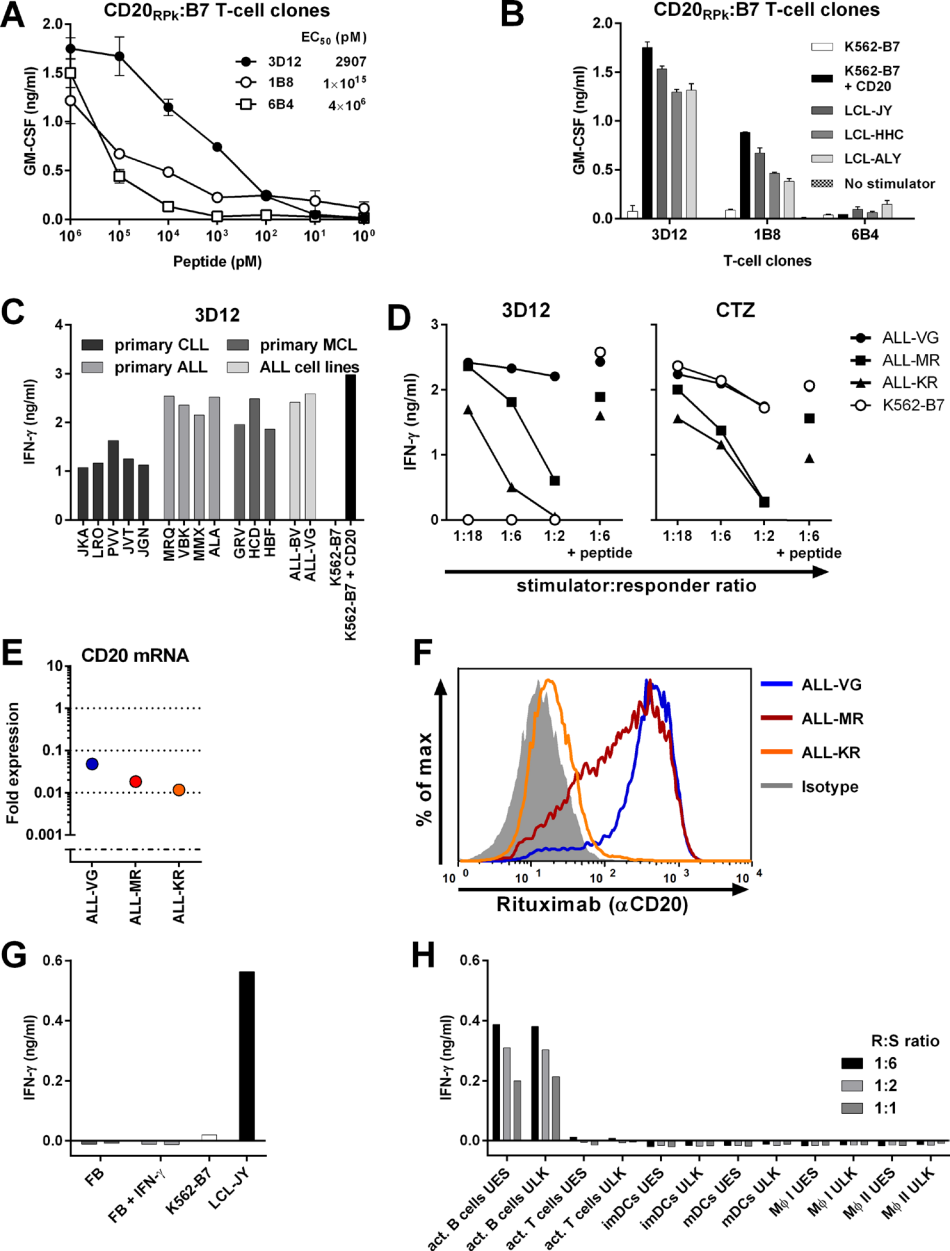
Identification of T-cell clone 3D12 recognizing CD20-derived peptide RPKSNIVLL in HLA-B7 T-cell clones 3D12, 1B8, and 6B4 were isolated from two healthy HLA-B7^neg^ individuals using pMHC-tetramers composed of CD20-derived peptide RPKSNIVLL (CD20_RPK_) bound to HLA-B7. (**A**) Clones 3D12, 1B8, and 6B4 were coincubated with CD20-negative K562 cells expressing HLA-B7 (K562-B7) that were exogenously loaded with titrated amounts of peptide CD20_RPK_. (**B**) Clones 3D12, 1B8, and 6B4 were cocultured with K562-B7 cells, K562-B7 cells transduced to express CD20 (+ CD20) or three naturally CD20-expressing HLA-B7^pos^ EBV-LCLs. Shown are means and standard deviations of one representative experiment carried out in duplicate. (**C**) Clone 3D12 was cocultured with primary HLA-B7^pos^ B-cell malignancies and ALL cell lines. Malignant cell samples include chronic lymphocytic leukemia (CLL), acute lymphoblastic leukemia (ALL) and mantle cell lymphoma. (**D**) Clone 3D12 was cocultured with 3 HLA-B7^pos^ ALL cell lines at indicated responder-to-stimulator ratios. Peptide-pulsed stimulators (1:6 + peptide) served as positive control. T-cell clone CTZ recognizing a ubiquitously expressed antigen in the context of HLA-B7 served as positive control. (**E**–**F**). Expressions of CD20 mRNA in ALL cell-lines was measured by RT-qPCR and is shown as fold expression of average expression in healthy B-cells which was set to 1. The detection limit of the assay was > 0.001 fold (E). Cell surface CD20 on ALL cell-lines was assessed by FITC-conjugated CD20-specific monoclonal antibody rituximab **(**F). Controls included staining with an isotype-matched antibody (gray area). (**G**–**H**) Clone 3D12 was cocultured with nonhematopoietic and hematopoietic cell subsets. Fibroblast (FB) of two HLA-B7^pos^ individuals were cultured in the absence or presence of IFN-γ (+ IFN-γ) for 4 days prior to coculture with clone 3D12 (G). Clone 3D12 was cocultured with activated hematopoietic cell subsets of two healthy donors at different responder-to-stimulator (R:S) ratios (H). The stimulatory capacity of stimulator cells was verified by a control clone. Representative data from one of two independent experiments. GM-CSF and IFN-γ secretion was assessed after 18 hours of coculture by standard ELISA. MΦ I and II, Macrophage type I and II, respectively.

An off-the-shelf TCR library targeting different CD20-derived peptides presented in various HLA alleles could broaden the applicability of CD20-specific TCRs. Therefore, we searched for other peptides derived from CD20 in our recently published data describing the HLA ligandome of B-lymphocytes [22]. This study investigated the peptidome of B-lymphocytes by eluting HLA-bound peptides from B-LCLs, and identifying and characterizing these peptides using tandem mass spectrometry. Matching tandem mass spectra of eluted and newly synthesized peptide verified correct identification of the HLA-B7 presented CD20-derived peptide RPKSNIVLL (CD20_RPK_; data not shown). Using the described high-throughput methodology, three CD20_RPK_-reactive T-cell clones were isolated from two HLA-B7^neg^ healthy individuals. From these three T-cell clones, clone 3D12 exhibited highest peptide-specific reactivity and strong recognition of three CD20-expressing HLA-B7^pos^ B-LCLs and K562-B7 cells transduced with CD20 (Figure 7A–7B). Furthermore, Clone 3D12 efficiently recognized primary HLA-B7^pos^ B-cell malignancies including ALL, CLL, and MCL despite different CD20 expression levels (Figure 7C and Supplementary Figure S4). Recognition of 5 CLL samples was comparable although CD20 mRNA expression was drastically reduced in CLL sample JGN by more than 100-fold compared to healthy B-cells. Similarly, clone 3D12 highly recognized ALL sample MMX although CD20 mRNA expression was reduced 40-fold compared to healthy B-cells. Recognition of 3 ALL cell-lines was robust (Figure 7D). Clone 3D12 efficiently recognized ALL-KR which had 100-fold reduced CD20 mRNA expression and almost entirely lacked cell surface CD20 (Figure 7E–7F). No reactivity towards healthy hematopoietic or nonhematopoietic cell subsets was observed (Figure 7G–7H).

These data demonstrated that our high-throughput approach can easily be employed to target additional peptides in different HLA alleles, and that HLA-B7-restricted CD20_RPK_-specific T-cells can readily recognize CD20^low^ B-cell malignancies.

## DISCUSSION

Therapeutic monoclonal antibodies (mAb) targeting the cell surface antigen CD20 have been successfully applied in the clinic. However, relapsed or refractory disease as a result of CD20 cell surface downregulation, internalization of the CD20:mAb complex and other mechanisms has been reported [3–10, 27–29]. Therefore, additional strategies to overcome these mechanisms of resistance are required. Here, we describe the isolation of high-affinity TCRs directed against CD20-derived peptides presented in the context of HLA class I.

To overcome tolerance for self-antigens such as CD20, T-cell clones were isolated from HLA-A2^neg^ or HLA-B7^neg^ individuals, thus exploiting the immunogenicity of allogeneic (non-self) HLA molecules to target self-antigens. Among the T-cell clones isolated using pMHC-tetramers, only a small fraction demonstrated sufficient peptide sensitivity and strong antigen-specificity. These results reflect our previous experiences when employing pMHC-tetramers to isolate T-cell clones [20, 30, 31].

T-cell clones were selected by binding to pMHC-tetramers and subsequently tested for their recognition of peptide-pulsed K562 cells. Although T-cell clones demonstrated CD20_SLF_-dependent recognition, many T-cell clones reacted to unloaded K562-A2 indicating recognition of other peptides presented in HLA-A2. Of note, reactivity to unloaded CD20-negative K562-A2 cells was an indication for harmful off-target toxicity which could be observed for promiscuous clone 1A5 when stimulated with fibroblasts (Supplementary Figure S7). Peptide promiscuity, i.e. reactivity of one TCR with several peptides, is not uncommon and has been described for several highly peptide-specific TCRs [32–34]. Furthermore, peptide promiscuity can span tens to thousands of different peptides for a given TCR, albeit with strongly varying degrees of peptide sensitivity for each peptide [33]. The ability of TCRs to interact with multiple peptide-HLA molecules is likely to stem from the necessity of the immune systems to cover all possible antigens with a relatively limited TCR repertoire within a single individual [35].

The T-cell clones were isolated from PBMCs of healthy individuals and could therefore stem from the naïve or differentiated effector and memory T-cell pool. To test whether differentiation or activation status of a clone could misguide the selection of candidate TCRs, the TCRs of different CD20-specific clones with varying peptide sensitivities were expressed on the same cellular background in virus-specific T-cells. TCR-transduced T-cells responded more sensitively towards exogenously loaded peptide if the introduced TCR was derived from a T-cell clone also requiring only low amounts of CD20 peptide, indicating that functional data obtained from each T-cell clone is a valid representation of the expressed TCR rather than intrinsic features of the T-cell clone.

T-cell clone 1E9 was selected because of highest peptide-sensitivity and stringent peptide-specificity. Efficient reactivity towards primary B-cell malignancies such as ALL, CLL and MCL was observed. Transfer of TCR-1E9 installed potent CD20-reactivity onto recipient T-cells and led to the lysis of primary B-cell leukemia. Moreover, lysis was also observed for ALL cell-lines that lacked sufficient cell surface CD20 to be susceptible to mAb-mediated CDC. Similarly, HLA-B7-restricted CD20-specific T-cell clone 3D12 recognized ALL-KR lacking almost entirely cell surface CD20. These data indicate a complementary role for CD20-specific TCRs in the treatment of B-cell malignancies. CD20-targeting mAbs are applicable irrespective of HLA genotype but are limited by the degree of cell surface CD20 and presence of secondary cytotoxic mediators such as CDC or immune cells. In contrast, our CD20-specific TCRs showed improved antigen-sensitivity and could be used to administer potent cytotoxic T-cells but are restricted to TCR gene therapy of HLA-A2^pos^ or HLA-B7^pos^ individuals. It could be argued that T-cells expressing CD20-specific chimeric antigen receptors (CARs) will be able to also target malignancies expressing low amounts of extracellular CD20. However, in a patient suffering from primary cutaneous marginal zone lymphoma with spleen involvement only a weak response of the splenomegaly was reported after treatment with CD20-specific CAR-engineered T-cells [36]. It was argued by the authors that very low CD20 expression on malignant cells as has been reported for some indolent splenic lymphoma patients [37] could have caused this weak response among other factors.

Furthermore, TCR-modified T-cells highly sensitive to CD20-derived peptides may prove especially useful in the treatment of CLL. Malignant cells of CLL patients generally express low levels of CD20. In addition, patients suffering from CLL frequently demonstrate complement deficiencies limiting the effectiveness of treatment with monoclonal antibodies [10]. Although CD19-targeting CAR-modified T-cells have been very effective in the treatment of ALL, they have been less successfully applied in the treatment of CLL [38]. Moreover, the emergence of CD19-antigen loss variants after CAR administration urges the development of additional strategies [39]. Therefore, CD20-specific TCRs open an additional avenue to target multiple antigens simultaneously to decrease the risk of tumor-escape variants.

No recognition of a panel of CD20-negative but HLA-A2^pos^ hematopoietic and nonhematopoietic cells was observed indicating a safe reactivity profile of our candidate T-cell clones 1E9 and 3D12. Nonetheless, potential reactivity towards untested cell subsets could exist. Furthermore, the higher sensitivity for CD20 of our TCR-1E9 and clone 3D12 could also increase the risk for on-target off-tumor toxicity that has not been observed for CD20-targeting mAbs. Therefore, to guard against adverse events that may occur in patients due to off-target or on-target off-tumor toxicities, TCR-modified T-cells should be additionally engineered with a suicide switch [40–47].

We demonstrate that a TCR library can be readily built using this approach as demonstrated by the isolation of T-cell clone 3D12 targeting an additional CD20-derived peptide in HLA-B7. This clone also highly recognized CD20^low^ B-cell malignancies in the absence of reactivity towards healthy hematopoietic and nonhematopoietic cell subsets. An off-the-shelf library of various high-affinity CD20-specific TCRs targeting various HLA alleles could significantly broaden the applicability of immunotherapy to a wider patient group.

In summary, we demonstrate that high-affinity CD20-specific TCRs raised from the allorepertoire can be a valuable addition to current treatment options of patients suffering from CD20^low^ B-cell malignancies by administering TCR-engineered T-cells with potent effector function.

## MATERIALS AND METHODS

### Culture conditions and cells

Peripheral blood was obtained from different individuals after informed consent. Peripheral blood mononuclear cells (PBMCs) were isolated using Ficoll-gradient centrifugation and were cryopreserved. T-cells were cultured in T-cell medium consisting of IMDM (Lonza, Basel, Switzerland) supplemented with 100 IU/ml IL-2 (Proleukine; Novartis Pharma, Arnhem, The Netherlands), 5% fetal bovine serum (FBS; Gibco, Life Technologies, Carlsbad, CA) and 5% human serum. Primary hematopoietic cell subsets were obtained from cryopreserved PBMCs of HLA-A*02:01^pos^ healthy donors that were incubated with either anti-CD4, anti-CD14, anti-CD19 or anti-CD34 magnetic microbeads (Miltenyi Biotec, Bergisch Gladbach, Germany) for 15 min at 4°C. Microbead-labeled cells were isolated on LS column (Miltenyi Biotec) according to manufacturer's protocol. Purity of isolated cells was assessed using FACS analysis and cells were only used in experiments if purity exceeded 95%. Immature and mature dendritic cells (DCs) were differentiated *in vitro* from isolated CD14^+^ cell populations as previously described [15]. Briefly, on day zero 1 × 10^6^ cells/ml were seeded in IMDM supplemented with, 100 ng/ml GM-CSF (Sandoz Novartis Pharma, Almere, The Netherlands), 500 IU/ml IL-4 (Schering-Plough, Kenilworth, NJ), and 10% human serum, and cultured for two days to obtain immature DCs. Mature DCs were generated by culturing immature DCs in IMDM supplemented with 100 ng/ml GM-CSF, 10 ng/ml TNFalpha (CellGenix, Freiburg, Germany), 10 ng/ml IL-1b (Bioscource Invitrogen, Camarillo, CA), 10 ng/ml IL-6 (Sandoz Novartis Pharma), 1 μg/ml PGE-2 (Sigma Aldrich, St. Louis, MO), 500 IU/ml INF-γ (Boehringer Ingelheim, Ingelheim am Rhein, Germany), and 10% human serum for an additional two days. Macrophages type I and II were *in vitro* differentiated from CD14^+^ monocytes. CD14^+^ monocytes were cultured for 8 days in IMDM containing 10% human serum in the presence of 50 ng/ml GM-CSF or 5 IU/ml CSF-1 (R&D Systems, Minneapolis, MN) to obtain Macrophages type I or II, respectively. Activated T-cells were generated by stimulating CD4^+^ and CD8^+^ T-cells with irradiated (35 Gy) feeders in a 1:5 ratio in T-cell medium supplemented with 0.8 μg/ml phytohemagglutinin (PHA; Biochrom AG, Berlin, Germany) for 10 days prior to experiment. Activated CD19^+^ B-cells were generated by coculturing CD19^+^ cells on CD40L-transduced irradiated (70 Gy) mouse-fibroblasts for 7 days in IMDM supplemented with 2 ng/ml IL-4 and 10% human serum. K562 cells expressing HLA-A2 (K562-A2) or HLA-B7 (K562-B7) were previously described [20, 48]. Acute lymphoblastic leukemia (ALL) cell-lines were derived from primary ALL cells and were previously described [49]. Fibroblasts were cultured from skin biopsies in Dulbecco's modified Eagle medium (DMEM; Lonza) containing 1g/l glucose and supplemented with 10% FBS as previously described [15]. Fibroblasts treated with IFN-γ were cultured in medium containing 200 IU/ml IFN-γ for four days prior to experiment. All cells were washed twice before use in experiments.

### Generation of peptide-MHC complexes

All peptides were synthesized in-house using standard Fmoc chemisty. Recombinant HLA-A2 or HLA-B7 heavy chain and human β_2_m light chain were in-house produced in *Escherichia coli*. Major histocompatibility complex (MHC) class I refolding was performed as previously described with minor modifications [50]. Peptide-MHC (pMHC) class I complexes were purified by gel-filtration using HPLC. pMHC-tetramers were generated by labeling biotinylated pMHC-monomers with streptavidine-coupled phycoerythrin (PE; Invitrogen, Carlsbad, CA) or allophycocyanin (APC, Invitrogen). Complexes were stored at 4°C.

### Isolation of CD20-reactive T-cell clones

T-cells binding to CD20-specific pMHC-tetramers composed of peptide CD20_SLF_ bound to HLA-A2 or peptide CD20_RPK_ bound to HLA-B7 were isolated from 250 to 1000 × 10^6^ PBMCs of healthy HLA-A2^neg^ or HLA-B7^neg^ individuals, respectively. PBMCs were incubated with PE-labeled pMHC-tetramers for 1 h at 4°C, washed twice, and incubated with anti-PE-microbeads (Miltenyi Biotec) for 15 min at 4°C. PE-labeled cells were isolated on an LS colomn (Miltenyi Biotec) according to manufacturer's instruction. Positively selected cells were stained with an Alexa700-labelled antibody against CD8 (Invitrogen/Caltag, Buckingham, United Kingdom) in combination with FITC-conjugated antibodies against CD4, CD14, and CD19 (BD Pharmingen, San Jose, CA). pMHC-tetramer^+^ CD8^+^ T-cells were single-cell sorted into round-bottom 96-well plates containing 5 × 10^4^ irradiated (35 Gy) feeders in 100 μl T-cell medium supplemented with 0.8 μg/ml PHA.

### High-throughput screen

Following 2 weeks of expansion, duplicates of the round-bottom 96-well plates containing the T-cell clones were made by resuspending and distributing T-cell medium containing the T-cell clones over two round-bottom 96-well plates using the Biomek 2000 workstation (Beckman Coulter, Brea, CA) operated through BioWorks software (Version 3.5; Beckman Coulter). Cells in one of the duplicate plates were washed five times before cell pellets were resuspended in 200 μl T-cell medium. Then, using the Biomek 2000 workstation, T-cell clones were distributed over flat-bottom 384-well plates by pipetting 20 μl of cell suspension per well. Subsequently, 20 μl of a cell suspension containing either peptide-pulsed or unloaded K562-A2 cells were added using the Biomek 2000 workstation. K562-A2 cells had been pulsed with peptide CD20_SLF_ by incubating K562-A2 cells in T-cell medium supplemented with 50 nM for 1 h at 37°C. After 18 h coincubation, supernatants were harvested and IFN-γ or GM-CSF production was measured by enzyme-linked immunosorbent assay (ELISA, Sanquin Reagents or R&D Systems, respectively) using the Biomek 2000 workstation.

### TCR gene transfer

TRAV and TRBV usage of T-cell clones was determined by reverse transcriptase PCR and sequencing using a previously established protocol [48]. V(D)J segments of the TCRα and TCRβ were codon optimized and cloned into the modified MP71-TCR-flex retroviral backbone. To increase expression and preferential pairing of the introduced TCRαβ chain, the MP71-TCR-flex vector contains codon-optimized and cysteine-modified murine TCRαβ constant domains and porcine teschovirus-derived P2A sequence to link TCR chains [26]. Constructs were ordered from GenScript (Piscataway, NJ).

Virus-specific T-cells were generated by MACS isolation using pMHC-tetramers and stimulated with irradiated feeders and PHA [51]. Purified CD8^+^ T-cells were activated using irradiated autologous PBMCs and PHA. On day 2 following stimulation of T-cells, retroviral supernatant was loaded on 24-well nontissue culture–treated plates that had been coated with 30 mg/mL retronectine (Takara, Shiga, Japan) and blocked with 2% human serum albumin (Sanquin Reagents, Amsterdam, The Netherlands). Viral supernatant was spun down at 2000 *g* for 20 minutes at 4°C before activated T-cells were added to retroviral supernatant and incubated at 37°C for 18 hours. Cells were transferred to culture-treated plates containing fresh T-cell medium. Seven days after stimulation, high-purity TCR-transduced T-cells were obtained by MACS isolation based on the expression of the transduced TCR or marker gene NGF-R. Transduced T-cells were incubated with an APC-labelled antibody against the murine constant TCR domain (BD Pharmingen) or nerve growth factor-receptor (NGF-R or CD271, Sanbio, Uden, The Netherlands) for 15 min at 4°C and washed twice. Following incubation with anti-APC microbeads (Miltenyi Biotec) for 15 min at 4°C, TCR-transduced T-cells were isolated on a LS column following manufacturer's instructions.

### FACS analysis

FACS was performed on a LSRII (BD Biosciences, Franklin Lakes, NJ) or a FACS Calibur (BD Biosciences) and analyzed using Diva Software (BD Biosciences) or FlowJo Software (TreeStar, Ashland, OR). 10,000 cells of a T-cell clone were mixed with 10,000 CD4^+^ T-cells from third party to prevent aggregate formation and stained with 2 μg/ml PE- or APC-labelled pMHC-tetramers for 15 min at 37°C. An Alexa700-conjugated antibody against CD8 (Invitrogen/Caltag) combined with fluorescein isothiocyanate (FITC)-labelled antibodies against CD4, CD14, and CD19 (BD Pharmingen) was added for an additional 15 min at 4°C. Similarly, 25,000 TCR-transduced or mock-transduced T-cells were first incubated with pMHC-tetramers before antibodies against CD8, CD4 and NGF-R were added. PBMCs, purified hematopoietic cell subsets or activated cells were stained with antibodies against CD3, CD4, CD14, CD19, CD34 (BD Pharmingen) for 4°C for 15 min. To analyze the CD20 expression, 50,000 cells of an ALL cell-line were incubated with FITC-conjugated rituximab at a final concentration of 10 μg/ml or an IgG1 isotype control antibody at 4°C for 15 min. To generate FITC-conjugated rituximab, unlabeled rituximab was incubated with FITC isomer I (Sigma-Aldrich) in 0.5 M sodium carbonate buffer (pH 9.5) for 1 hour at room temperature. FITC-conjugated rituximab was purified by filtration using 3kD VivaSpin 20 centrifugal concentrators (Vivaproducts, Littleton, MA) spun at 2750 *g.* During filtration, sodium carbonate buffer was exchanged for by adding tris buffered saline (TBS, pH 8.2; Sigma Aldrich). FITC-conjugated rituximab was stored at 4°C.

### Functional analysis

Stimulator cells were peptide-pulsed at various peptide concentrations for 30 min at 37°C. Responder T-cells and peptide-pulsed or unloaded stimulator cells were coincubated at various responder to stimulator ratios. After 18 h coincubation, supernatants were harvested and IFN-γ or GM-CSF production was measured by ELISA.

### Cr^51^ release assay

Standard Cr^51^ release assay was performed as previously described [52]. Briefly, target cells were labelled with 100 μCi Na_2_Cr^51^O_4_ for 1 hour at 37°C, washed three times, and added to effector cells in T-cell medium at various effector-to-target ratios to a final volume of 100 μl. Complement dependent cytotoxicity (CDC) was assessed by incubating Cr^51^-labelled target cells with human serum in the presence of CD20-specific mAb ofatumumab at various concentrations. Spontaneous and maximum release was measured by incubating target cells with medium alone and 1% Triton X-100, respectively. The tests were performed in triplicate. After 5 hours of coincubation, 25 μl of supernatant was harvested and analyzed on a MicroBeta 2450 Microplate Counter (Perkin Elmer, Waltham, MA). The percent specific lysis was calculated as follows: [(experimental release – spontaneous release)/(maximum release – spontaneous release)]×100%.

### FACS-based cytotoxicity assay

Adapted from Jedema *et al.* [53], 10,000 PKH26GL-labelled (Sigma-Aldrich) target cells were coincubated with T-cells at various effector:target ratios in 50 μl T-cell medium for 20 hours. After coincubation, cells were stained with Sytox Blue dead cell stain (Invitrogen/Caltag) in a final concentration of 1 μM for 5 min. 10 μl Flow-count fluorospheres (Beckman Coulter) were added and samples were analyzed using FACS. For each sample, 3,300 Flow-count fluorospheres were acquired and the percent surviving cells was calculated as follows: (PKH26GL-labelled targets in the presence of effector cells)/(PKH26GL-labelled targets in absence of effector cells)×100%.

### Real-time quantitative PCR (RT-qPCR) of CD20

Total RNA was isolated using the RNAqueous Micro-Kit and Small Scale Kit (Ambion, Life Technologies) for a maximum of 0.5 × 10^6^ and 10 × 10^6^ cells, respectively, following the manufacturer's instructions. Total RNA was converted to cDNA using M-MLV reverse transcriptase (Invitrogen). *MS4A1* expression (coding gene of CD20) was measured on the Roche Lightcycler 480 (Roche, Basel, Switzerland) using Fast Start TaqDNA Polymerase (Roche) and EvaGreen (Biotium, Hayward, CA) with forward primer 5′-GGGGCTGTCCAGATTATGAA-3′ and reverse primer 5′-GGAGTTTTTCTCCGTTGCTG-3′. mRNA expression of *MS4A1* in samples was calculated using the 2^−ΔΔCT^ method [54] with the expression of *PBGD* serving as endogenous reference gene and the average expression of *MS4A1* in 7 healthy CD19^+^ B-cell samples serving as calibrator.

## SUPPLEMENTARY MATERIALS TABLES AND FIGURES


