# Supplementary material for: Generation of CD20-specific TCRs for TCR gene therapy of CD20low B-cell malignancies insusceptible to CD20-targeting antibodies
Source: Oncotarget. 2016 Oct 20;7(47):77021–37. doi: 10.18632/oncotarget.12778 (PMC5363567; doi:10.18632/oncotarget.12778)
Supplement: Supplementary file 1 [file oncotarget-07-77021-s001.pdf]

Generation of CD20-specific TCRs for TCR gene therapy of CD20^{low} B-cell malignancies insusceptible to CD20-targeting antibodies

Supplementary Materials

Supplementary Table S1: Total number of T-cell clones screened per HLA-A2^{neg} healthy donor and distribution of T-cell clones over three different reactivity profiles depicted in Supplementary Figure S1A

	Number of T-cell clones screened	T-cell clones not producing cytokines (%)	T-cell clones reactive with unloaded K562-A2 cells (%)	T-cell clones selected for further assessment (%)
Donor 1	499	22.8	73.8	3.4
Donor 2	331	52.6	37.8	9.6
Donor 3	1605	61.4	33.1	5.5
Donor 4	95	43.2	53.7	3.1
Donor 5	1031	65.1	28.1	6.8
Donor 6	71	56.4	39.4	4.2

This data is based on GM-CSF secretion as readout parameter

Supplementary Table S2: HLA typing of B-LCL panel used in this study (Adapted from van Loenen *et al.* [25])

	HLA class I			HLA class II		
**B-LCL**	**A**	**B**	**C**	**DR**	**DQ**	**DP**
EBM	23	64	8	4	8	1*0201
NGZ	11, 24	53, 61	2	1, 11	5, 7	1*0201, 1*0301
LMB	29	44, 51	14, 16	7, 8	2, 4	1*0401, 1*110101
GGT	26, 31	14, 49	7, 8	1, 7	2, 5	1*0402, 1*110101
IZA	2, 24	8, 60	3, 7	13, 17	2, 6	1*0401, 1*1401
MHX	1, 0205	18, 50	6, 7	7, 9	2, 9	1*0201, 1*0301
FAQ	23, 68	14, 38	8, 12	13	7, 6	1*0201
MBX	1	8, 63	7	12, 17	2, 7	1*0101, 1*0401
CVV	11, 31	57, 62	3, 6	4, 7	7, 9	1*0201, 1*0401
NGI	11, 24	8, 39	7	17, 8	2, 4	1*0101, 1*1401
AHT	24, 25	55, 62	3	8, 15	5, 6	1*0401
LSR	32, 68	35, 52	12	15, 16	5, 6	1*0401, 1*1401
ABV	3, 29	7, 44	7	7, 14	5, 2	1*0401, 1*1101
MWX	1, 34	15, 35	4, 12	1, 15	5, 6	1*0601, 1*1301
AKB	1, 2	37, 39	6, 7	1, 10	5	2*0102, 1*0401
UKL*	1, 30	8, 13	6, 7	4, 17	2, 7	1*0401
UCE*	3, 11	7, 27	2, 7	11, 14	5, 7	1*0201, 1*1601
RBB*	2, 26	8, 27	1, 7	17, 52	2	1*0401

*B-LCL not used in previous study and added in this panel.

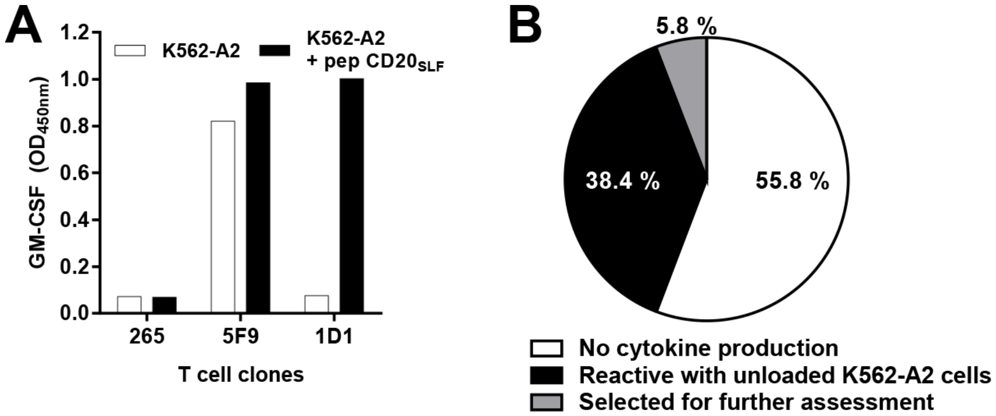

Supplementary Figure S1: Selection of candidate T-cell clones after pMHC-tetramer guided enrichment. $CD8^+$ T-cell clones were isolated from HLA-$A2^{neg}$ healthy individuals by binding to pMHC-tetramer composed of CD20-derived peptide SLFLGILSV bound to HLA-A2 ($CD20_{SLF}$:A2) and clonally expanded. (**A**) Shown are 3 representative clones tested in a first high-throughput screening. T-cells were coincubated with $CD20^{neg}$ K562 cells that stably expressed HLA-A2 (K562-A2) either in the absence or presence of 50 nM peptide $CD20_{SLF}$. (**B**) Distribution of T-cell clones over the different reactivity profiles observed in A. This data is based on GM-CSF secretion as readout parameter. Pie chart represents pooled data from all 6 HLA-$A2^{neg}$ healthy individuals.

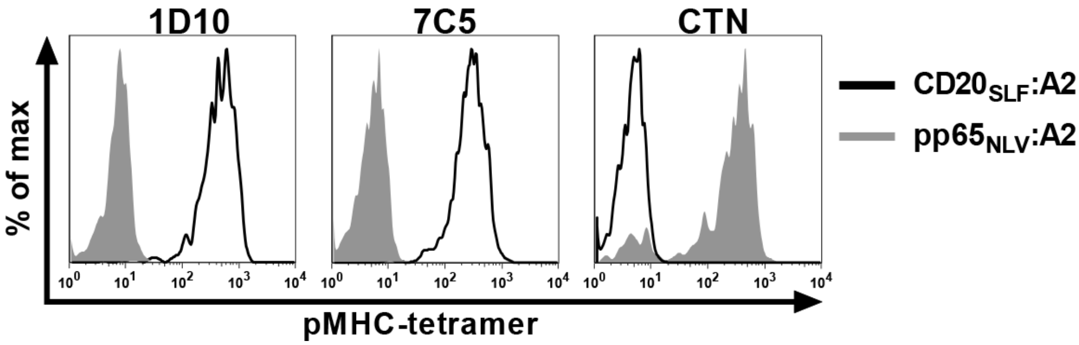

Supplementary Figure S2: pMHC-tetramer stainings of additional T-cell clones selected following high-throughput screening. Shown are histograms of T-cell clones that were stained either with pMHC-tetramer composed of $CD20_{SLF}$:A2 (black line) or a control pMHC-tetramer composed of CMV-derived peptide $pp65_{NLV}$ bound to HLA-A2 ($pp65_{NLV}$:A2, grey area). Controls included T-cell clone CTN specific for pMHC-tetramer $pp65_{NLV}$:A2.

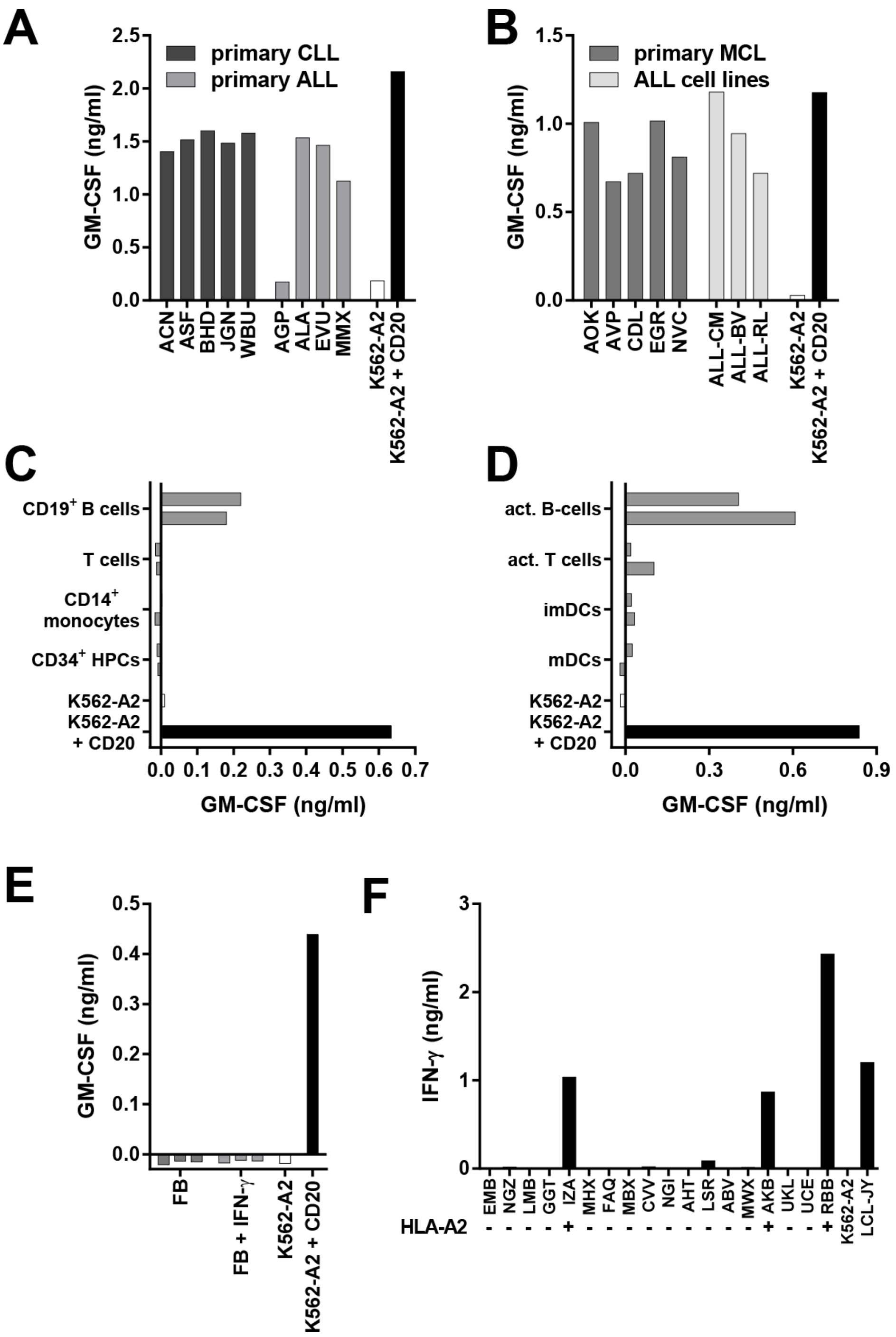

Supplementary Figure S3: CD20-reactive T-cell clone 28 recognizes B-cell malignancies while sparing healthy cell subsets. T-cell clone 28 was isolated using pMHC-tetramer composed of peptide $CD20_{SLF}$ bound to HLA-A2. (**A–B**) The same cells as in Figure 3A–3B were used. T-cell clone 28 was cocultured with HLA-$A2^{pos}$ primary B-cell malignancies or ALL cell-lines. Primary samples included chronic lymphocytic leukemia (CLL) and acute lymphoblastic leukemia (ALL) (A) or primary mantle cell lymphoma (MCL) (B). Primary ALL sample AGP did not demonstrate CD20 expression at the mRNA level (Supplementary Figure S4). Controls included $CD20^{neg}$ K562-A2 and CD20-transduced K562-A2 (K562-A2 + CD20). Culture supernatant was harvested after 18 hours of coincubation and cytokine production was assessed by standard ELISA. Experiments were carried out in duplicate. One representative experiment of two independent experiments. (**C**–**E**) The same cells as in Figure 5A-C were used. T-cell clone 28 was cocultured with different cell subsets of hematopoietic (C–D) or nonhematopoietic (E) origin of HLA-$A2^{pos}$ healthy individuals. Clone 28 was coincubated with primary (C) or activated (D) hematopoietic cell subsets from two different donors. Activated B-cells were generated by stimulating primary B-cell with CD40 ligand, activated T-cells were generated by stimulation with PHA. Immature and mature dendritic cells (imDCs and mDCs, respectively) were monocyte derived. T-cell clone 28 was coincubated with fibroblasts (FB) that had been cultured either in the absence or presence of 200 IU/ml IFN-γ (+IFN-γ) for 4 days prior to the experiment. (**F**) Clone 28 was coincubated with a panel of B-LCLs expressing different HLA class I and class II alleles. HLA status regarding presence (+) or absence (–) of HLA-A*0201 is indicated. For a complete list of HLA genotype for all B-LCL see Supplementary Table S2. After 18 hours of coincubation, supernatant was harvested and cytokine production was assessed using standard ELISA. Shown are representative results of two independent experiments.

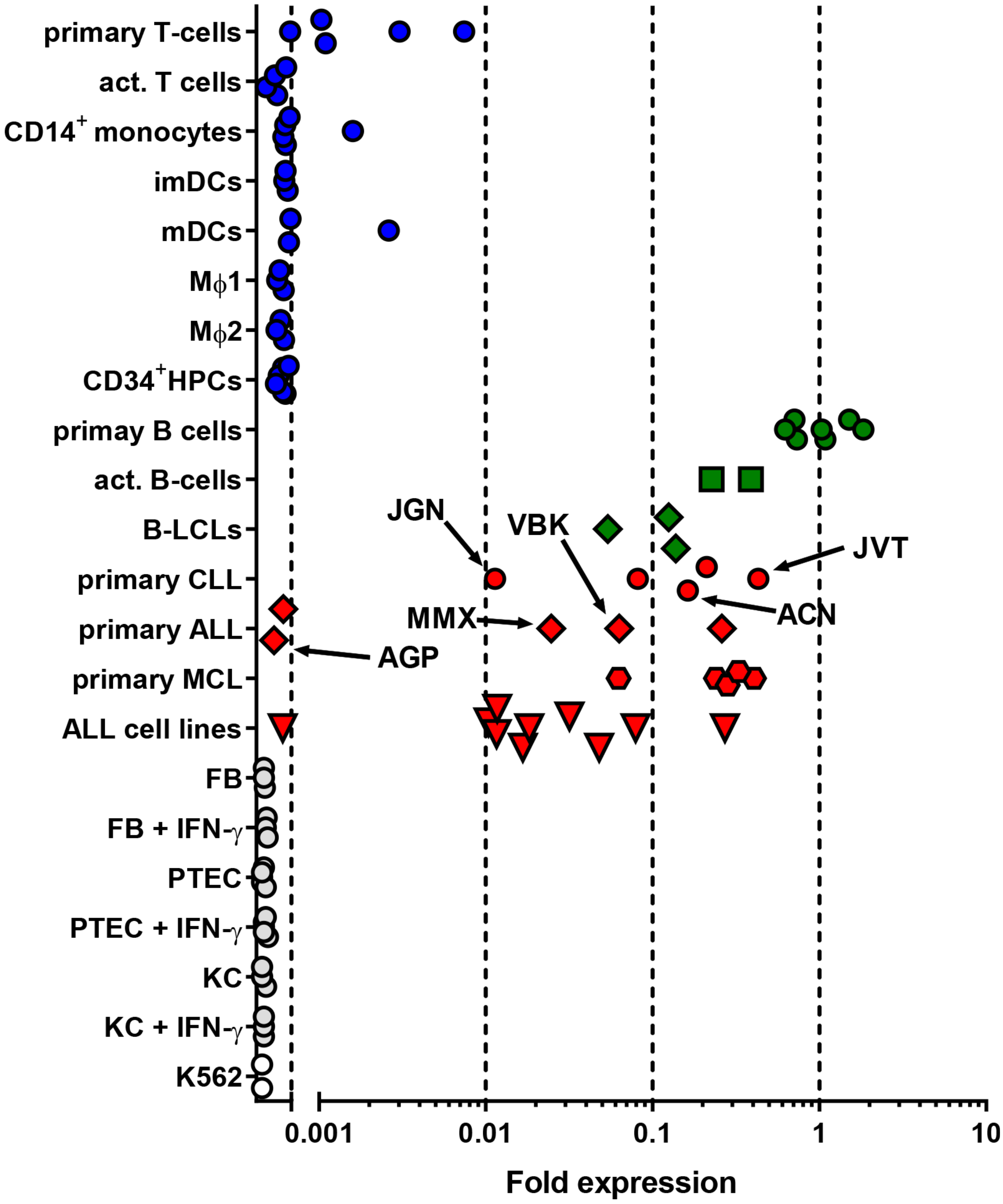

Supplementary Figure S4: CD20 mRNA expression in healthy and malignant hematopoietic and nonhematopoietic cell subsets. CD20 mRNA expression was measured by RT-qPCR. Shown are samples of healthy hematopoietic origin (blue dots), healthy B-cells (green dots), B-cell malignancies (red dots) and samples of nonhematopoietic origin (gray dots). Dots indicate individual samples. CD20 mRNA expression is shown as fold expression of average expression in 7 healthy B-cells which was set to 1. imDCs and mDCS, immature and mature dendritic cells; Mø1 and Mø2, macrophages type 1 and 2, respectively; HPCs, hematopoietic progenitor cells; B-LCL, EBV-transformed lymphoblastic B-cell lines; CLL, chronic lymphocytic leukemia; ALL, acute lymphoblastic leukemia; FB, fibroblasts; PTEC, proximal tubular epithelia cells; KC, keratinocytes; +IFN-γ, cells were cultured in medium supplemented with IFN-γ. Arrows indicate cell samples mentioned in the text. The detection limit of the assay was > 0.001 fold.

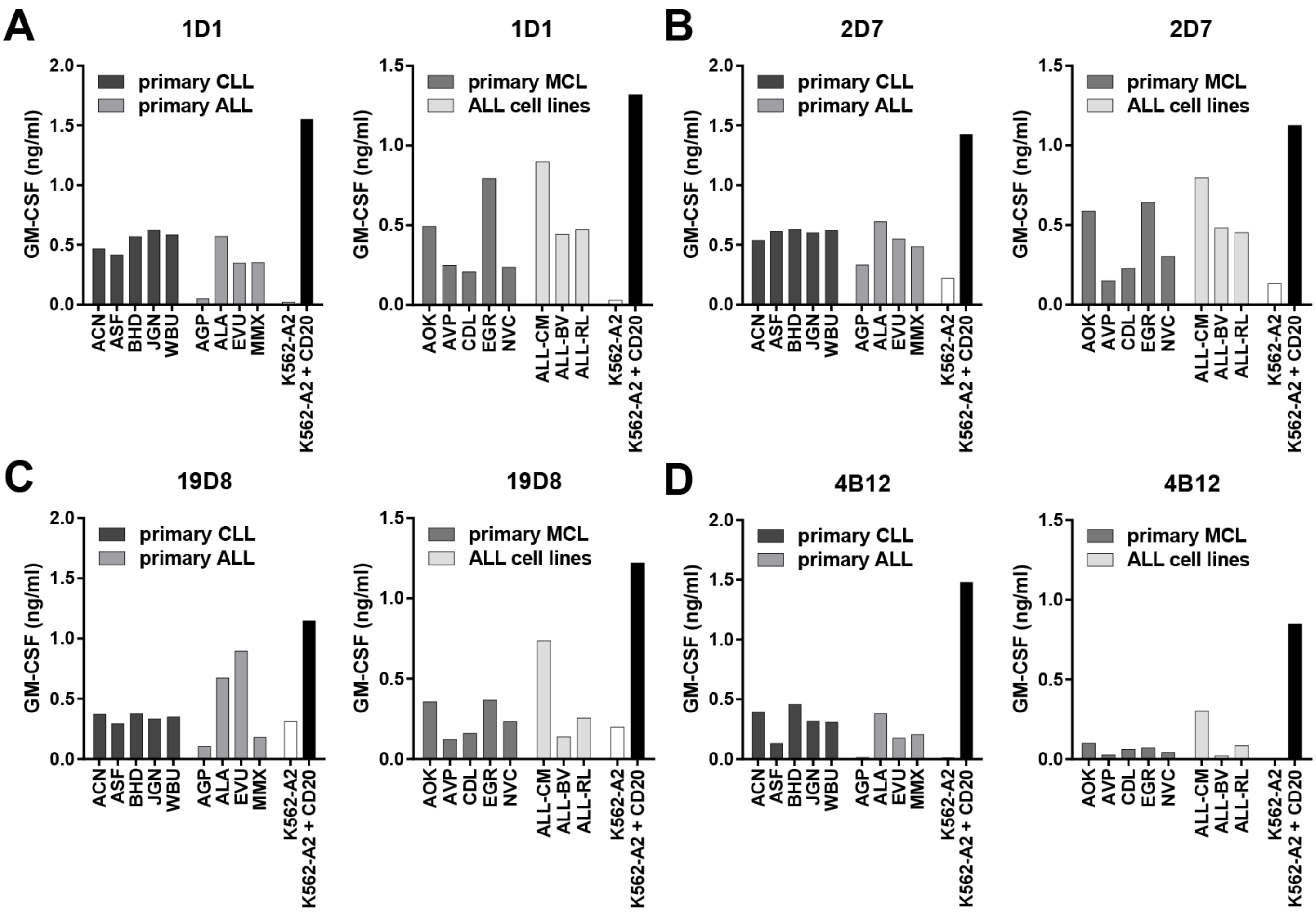

Supplementary Figure S5: T-cell clones 1D1, 2D7, 19D8 and 4B12 show different degrees of recognition of primary B-cell malignancies. (**A–D**) T-cell clones 1D1 (A), 2D7 (B), 19D8 (C), or 4B12 (D) were cocultured with the same HLA-$A2^{pos}$ primary B-cell malignancies as in Figure 3A–3B. Controls included $CD20^{neg}$ K562-A2 cells and CD20-transduced K562-A2 (K562-A2 + CD20). IFN-γ production was assessed after 18 hours of coincubation. Shown are means of one experiment carried out in duplicate.

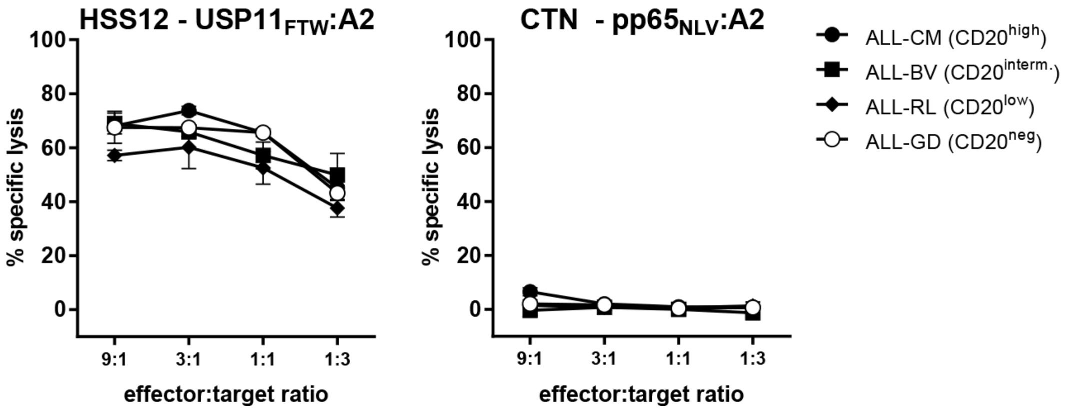

Supplementary Figure S6: Control experiments for Cr^{51}-release assay. The same target cells as in Figure 3D were used. Cr^{51}-labelled targets were incubated with clone HSS12 or a CMV-reactive clone CTN at different effector-to-target ratios for 5 hours. Targets included HLA-$A2^{pos}$ ALL cell-lines ALL-CM, ALL-BV, ALL-RL and ALL-GD. HLA-$A2^{neg}$ ALL cell-line ALL-KW served as a negative control. T-cell clone HSS12 recognizes peptide FTWEGLYNV from the ubiquitously expressed gene USP11 in the context of HLA-A2. CMV-reactive T-cell clone CTN recognizes the irrelevant pp65-derived peptide NLVPMVATV in the context of HLA-A2. Shown are means with standard deviations of one experiment performed in triplicate.

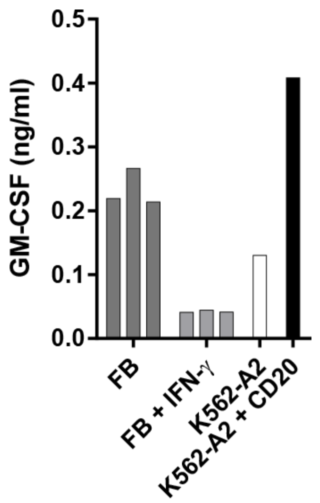

Supplementary Figure S7: Recognition of an irrelevant peptide other than $CD20_{SLF}$ leads to off-target reactivity of T-cell clone 1A5. T-cell clone 1A5 was coincubated with the same fibroblast (FB) samples as in Figure 4C. Fibroblasts were left untreated or cultured in medium containing 200 IU/ml IFN-γ (+IFN-γ) for 4 days prior to coincubation. GM-CSF production was assessed after 18 hours of coincubation.
